# Correlation between CBC-derived inflammatory indicators and all-cause mortality with rheumatoid arthritis: a population-based study

**DOI:** 10.3389/fmed.2025.1538710

**Published:** 2025-06-10

**Authors:** Yu Liu, Yiping Liu, Shao Fan, Jing Yang, Mingxi Xu, Lin Zhao, Changyan Liu, Yida Xing, Xiaodan Kong

**Affiliations:** ^1^Department of Rheumatology, The Second Affiliated Hospital of Dalian Medical University, Dalian, China; ^2^Dalian Key Laboratory of Autoantibody Detection, The Second Affiliated Hospital of Dalian Medical University, Dalian, China; ^3^College of Basic Medical Sciences, Dalian Medical University, Dalian, China

**Keywords:** rheumatoid arthritis, mortality, NHANES, systemic inflammatory response index, neutrophil-to-lymphocyte ratio, monocyte-to-lymphocyte ratio

## Abstract

**Objectives:**

We investigated the relationship between inflammatory indicators derived from complete blood cell (CBC) counts and all-cause mortality in individuals with rheumatoid arthritis (RA).

**Methods:**

Data were collected from the National Health and Nutrition Examination Survey (NHANES) database from 2007 to 2018, with a median follow-up duration of 78 months. The inflammatory indicators derived from CBC included several types: the systemic inflammatory response index (SIRI), the systemic immune-inflammation index (SII), the neutrophil-to-lymphocyte ratio (NLR), the platelet-to-lymphocyte ratio (PLR), and the monocyte-to-lymphocyte ratio (MLR). The multiple COX regression models were used to estimate adjusted hazard ratios (HRs) and 95% CIs concerning all-cause mortality of participants with RA, which focused on CBC-derived inflammatory indicators. Additionally, restricted cubic spline (RCS) curve was utilized to investigate non-linear associations.

**Results:**

The research comprised a cohort of 1,314 individuals, among whom 246 with RA succumbed during a median follow-up duration of 78 months. After adjusting for key covariates, the mortality rate in patients with RA who had high SIRI, NLR, and MLR levels was considerably higher than in those with medium or low SIRI, NLR, and MLR levels. Compared with the lowest tertile, the highest tertiles of SIRI (HR 1.87, 95% CI: 1.12–3.13), NLR (HR 1.79, 95% CI: 1.10–2.92), and MLR (HR 1.88, 95% CI: 1.17–3.02) were associated with an increased risk of all-cause mortality. The Kaplan-Meier analysis indicated a significant decrease in the survival probability among individuals with elevated SIRI, NLR, and MLR levels. The RCS analysis revealed a linear association between SIRI, NLR, MLR, and RA-related all-cause mortality, whereas a non-linear relationship was identified between the SII, PLR, and mortality.

**Conclusion:**

This investigation revealed that the SIRI, NLR, and MLR are novel, valuable, and convenient inflammatory indicators. In the United States adults with RA, higher SIRI, NLR, and MLR were independently associated with an increased long-term mortality risk. These findings not only assist in uncovering the potential utility of predicting RA outcomes but also provide rheumatologists valuable guidance for disease management.

## 1 Introduction

Rheumatoid arthritis (RA) is a chronic inflammatory disorder with excessive synovial tissue growth, pannus formation, and gradual bone and cartilage deterioration (1). Undoubtedly, RA has a high prevalence rate and continues to pose a significant global public health issue. Worldwide, the disease burden of RA has increased and will persist in growing. Prevention and early intervention are crucial in preventing disease flares and reducing the enormous burden associated with RA (2). Predictions indicate that the burden of RA will keep on increasing to the extent that the global prevalence of disease will reach 31.7 million by 2050 (3). Identifying the impacts influencing RA morbidity and mortality is essential for developing effective management strategies and interventions. The observed increase in RA mortality rates during the follow-up period enhances our comprehension of disease progression and its consequences (4). It is well-established that specific autoantibodies in RA play a notable role in diagnosing disease activity and predicting prognosis (5). However, recent research has shifted focused toward inflammatory indicators for disease evaluation and prognosis (6). Multiple researches have emphasized the crucial role of inflammation in the progression and pathogenesis of RA (7). While Inflammatory indicators are commonly used for RA diagnosis and disease assessment, there is a lack of data about their association with mortality. Moreover, novel inflammatory indicators derived from routine blood tests have shown potential for monitoring RA-related mortality and aiding in disease management. Consequently, rheumatologists are actively seeking other accessible inflammatory indicators that can be utilized to manage and evaluate the entire course of RA.

Recent studies have reported notably elevated levels of inflammatory indicator in RA patients compared to healthy controls. Furthermore, these inflammatory indicator levels, derived from complete blood analysis results, have proven to be easily obtainable and cost-effective indicators of RA disease activity. The biomarkers include neutrophils, lymphocytes, platelets, and monocytes. In addition, inflammatory indicators derived from CBCs, such as the systemic inflammatory response index (SIRI), systemic immune-inflammation index (SII), neutrophil-to-lymphocyte ratio (NLR), platelet-to-lymphocyte ratio (PLR), and monocyte-to-lymphocyte ratio (MLR), are implicated in various diseases (8). These indicators are crucial inflammatory cells that secrete cytokines, chemokines, proteases, and angiogenic factors during chronic inflammation (9). Numerous composite blood scores have been proposed to evaluate disease activity for this purpose. Recently, several studies have been demonstrated a correlation between the NLR and PLR with RA disease activity (10). Nonetheless, it exists a gap in the literature concerning a systematic and comprehensive investigation of the association between CBC-derived inflammatory indicators in RA patients and all-cause mortality.

Overall, research has established a connection between inflammatory indicators derived from CBC and the manifestation of RA in individuals (11). The SIRI has demonstrated potential as a non-invasive and effective biomarker for diagnosing and evaluating RA activity, predicting RA-associated interstitial lung disease (RA-ILD), and assessing tumor development (12). Similarly, the SII is recognized as a novel inflammatory marker associated with RA disease activity (13). Commonly, inflammatory indicators derived from CBCs, such as the erythrocyte sedimentation rate (ESR), C-reactive protein (CRP), and Disease Activity Score 28 (DAS28), are utilized to evaluate RA activity (14). The NLR is an emerging biomarker that provides insights into immune system activation and systemic inflammation (15). Consequently, an elevated NLR serves as a reliable and cost-effective prognostic indicator of cardiovascular and overall mortality in individuals with RA (16). Additionally, the PLR can be used as a potential marker of systemic bone loss in patients with RA (17). The MLR may be employed as a supplementary diagnostic indicator in patients with undifferentiated inflammatory arthritis (18). For now, several articles have reported links between CBC-derived inflammatory indicators and mortality in patients with conditions such as psoriatic arthritis (19) and asthma (20). However, several investigations have the limitation of small sample sizes. Although a limited number of papers have explored the correlation between inflammatory indicators derived from CBC and the activity and morbidity associated with RA, there is a paucity of research on the relationship between these Inflammatory indicators and the survival outcomes of RA patients. Identifying independent risk factors related to the survival status of RA patients is crucial for effective disease management and prognosis assessment.

To date, the potential association between inflammatory indicators identified in CBCs, using data from the National Health and Nutrition Examination Survey (NHANES), and all-cause mortality in RA patients remains largely unexplored. From 2007 and 2018, an analysis was conducted involving 1,314 individuals with RA to investigate the associations between SIRI, SII, NLR, PLR, and MLR with all-cause mortality. Our investigation aimed to identify a cost-effective and readily available prognostic indicator for individuals with RA.

## 2 Materials and methods

### 2.1 Study design and population

**FIGURE 1:**
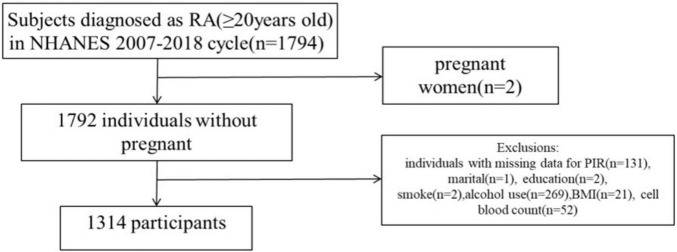
National Health and Nutrition Examination Survey (NHANES) 2007–2018 study flow chart.

Using NHANES, the National Center for Health Statistics evaluates the nutritional and health conditions of non-institutionalized civilians. The NCHS Institutional Review Board granted approval to the NHANES proposal, and all survey participants have provided informed consent and signed a consent form. This study included 1,794 persons diagnosed with RA during six consecutive cycles from 2007–2008 to 2017–2018. Among these individuals, two were pregnant and 478 had missing data on core covariates (131 PIR deletions, one marital status deletion, two education status deletions, two smoking status deletions, 269 alcohol use deletions, 21 BMI deletions, and 52 cell blood count deletions). In summary, the case study ultimately included a total of 1,314 participants (Figure 1).

### 2.2 A comprehensive evaluation of RA

Individuals’ circumstances were assessed using self-report questionnaire. The survey comprises three inquiries: Have you ever received a diagnosis of arthritis from a doctor or any other healthcare professional? At what age were you initially diagnosed with arthritis? What is the specific type of arthritis? Based on the aforementioned three screening questions, participants who had received a diagnosis of RA were selected for inclusion in the assesses. It has been reported that the coincidence rate between self-reported diagnosis and clinical diagnosis is more than 85% (7, 21, 22).

### 2.3 Evaluation of inflammatory indicators generated from CBC

By measuring the whole blood cell count using an automated hematology analyzer, various blood components, including red blood cells, white blood cells, and platelets, were quantified for the given volume of blood. Below are formulas for the CBC-derived inflammatory indicators:


$$\mathrm{SIRI}=\frac{\mathrm{neutrophil}\,\mathrm{count}*\mathrm{monocyte}\,\mathrm{count}}{\mathrm{lymphocyte}\,\mathrm{count}}$$

$$\mathrm{SII}=\frac{\mathrm{neutrophil}\,\mathrm{count}*\mathrm{platelet}\,\mathrm{count}}{\mathrm{lymphocyte}\,\mathrm{count}}$$

$$\mathrm{NLR}=\frac{\mathrm{neutrophil}\,\mathrm{count}}{\mathrm{lymphocyte}\,\mathrm{count}}$$

$$\mathrm{PLR}=\frac{\mathrm{platelet}\,\mathrm{count}}{\mathrm{lymphocyte}\,\mathrm{count}}$$

$$\mathrm{MLR}=\frac{\mathrm{monocyte}\,\mathrm{count}}{\mathrm{lymphocyte}\,\mathrm{count}}$$

### 2.4 Covariates

Factors that could confound the association between inflammatory indicators obtained from CBCs and mortality in RA were carefully managed, taking into account the literature and pertinent clinical expertise. The primary covariates are listed as follows: According to the World Health Organization classification of age, the age variable was divided into two groups: young (under 60 years) and elderly (60 years and older). There are two categories for gender: male and female. Four categories exist for race: Mexican-Americans, non-Hispanic blacks, non-Hispanic whites and other. Divorced, separated, or never married are the various categories of marital status. Three categories make up the poverty-income ratio (PIR): The low-income group includes people or families with incomes of 1.35% or less; the moderate group includes people or families with incomes of 1.35%–3.5%; and the high group includes people or families with incomes of 3.5% or more. There are two categories of body mass index (BMI): normal (BMI < 25.0 kg/m^2^) and obese (BMI ≥ 25.0 kg/m^2^). Alcohol intake is divided into two categories: never (less than 12 drinks in a person’s lifetime) and ever/current (12 or more drinks in a person’s lifetime). In terms of smoking status, there are two categories: never smokers and former/current smokers (≥ 100 cigarettes in a lifetime). It is possible to diagnose hypertension based on the self-reported medical history, blood pressure readings greater than 140/90 mm Hg annually, or the use of antihypertensive medications. Having an HbA1c level of at least 6.5%, a random blood glucose level of at least 11.1 mmo1/L, a fasting plasma glucose level of at least 7.0 mmo1/L, or self-reported use of insulin or other diabetes medications indicates that an individual has diabetes.

### 2.5 Statistical analysis

To accurately represent the Correlation between the selected samples and the actual population, and to mitigate the effects of missing samples, oversampling, and differences in sample selection on the overall analysis, we employed a complex sampling analysis method and applied weight to the samples. The population was stratified into tertiles based on the indicators under study within the included population. Kaplan-Meier curves were generated to visually depict the survival probabilities associated with several categories of inflammatory indicators derived from complete blood counts. Cox proportional hazards models, calculated using both univariate and multivariate weights, were utilized to analyze the correlation between inflammatory indicators from CBC and all-cause mortality in RA patients. Three distinct models were constructed: Model 1 adjusts only for age and gender; Model 2 incorporated additional considers for race/ethnicity, marital status, poverty-to-income ratio, and body mass index; Model 3 further adjusted for factors such as diabetes, hypertension, smoking status, drinking status, among others. To investigate whether there was a non-linear relationship between all-cause mortality from RA and inflammatory biomarkers collected from CBC, we used restricted cubic spline (RCS) analysis. The entire statistical analysis was conducted by R statistical software, specifically version 4.3.1.

## 3 Results

### 3.1 Characteristics of the RA participants

Based on the information for SIRI, SII, NLR, PLR, and MLR ternaries presented in Supplementary Table 1, the study population demonstrated the following weighted demographic baseline characteristics. The data in Supplementary Table 1a indicate that the high SIRI group was significantly overrepresented by male patients compared to the low and medium SIRI groups. Additionally, the high SIRI subgroup had a higher percentage of adults aged 60 and above as well as a greater percentage of individuals with hypertension. According to Supplementary Table 1b, the prevalence of hypertension was notably greater among patients in the high SII subgroup compared to those in the other two subgroups. Supplementary Table 1c reveals that the population with a high NLR had a greater proportion of individuals aged 60 years and older contrasted with the low and middle NLR groups, and the prevalence of hypertension was similarly elevated in the high group. Supplementary Table 1d highlights a notable disparity in the number of females between the high PLR population and the low and medium PLR populations. Furthermore, the number of non-drinkers in the high PLR group was significantly higher than those in the low and medium PLR groups. Supplementary Table 1e suggests that with the increase in MLR, there was a noticeable increase in the proportion of males, people over 60 years old, and persons with hypertension.

### 3.2 Measuring and predicting RA mortality based on CBC-derived indicators

A 78 months follow-up was conducted (range: 1–157 months), 246 (18.75%) mortality events occurred. Cox proportional hazard analysis demonstrated a significant increase in mortality rates among participants with RA in the high SIRI group versus those in the low and medium SIRI groups (HR 2.58, 95% CI: 1.61–4.12). In Model 1, the high SIRI group had a statistically significant increase in all-cause mortality when age and sex were considered. This increase was also observed in comparison to the low and medium-SIRI groups (HR 2.17, 95% CI: 1.35–3.47). Model 2 demonstrated that, even after accounting for race/ethnicity, marital status, education, and PIR, the high-SIRI group exhibited a significantly greater all-cause death rate than did the low and medium SIRI groups (HR 2.01, 95% CI: 1.22–3.30).

After additional adjustments for diabetes, hypertension, smoking status, alcohol consumption, and BMI were made in Model 3, high SIRI patients had a significantly higher death rate from all causes than those with medium SIRI. Statistics indicated that this difference was significant in statistical terms (HR 1.87, 95% CI: 1.12–3.13). Patients with RA had a notable positive correlation between the SIRI and mortality. The relationship between SII and mortality in patients with RA was studied using a Cox proportional hazard model. According to the findings presented in Table 1, the crude model results indicate a statistically significant increase in all-cause mortality in the high-SII subgroup compared to the low and medium SII subgroups (HR 1.45, 95% CI: 1.04–2.02). According to Model 1, after adjusting for age and sex, the mortality rate of RA patients with a high SII remained significantly greater (HR 1.46, 95% CI: 1.07–2.00) than that of patients with a low or medium SII, and the difference was statistically significant. In Model 2 and Model 3, subsequent to making additional adjustments for race/ethnicity, marital status, education, PIR and diabetes status, hypertension status, smoking status, alcohol usage, and BMI, the all-cause mortality rate for RA patients in the high-SII subgroup remained greater than that in the low- SII subgroup and moderate-SII subgroup. However, the difference was no longer statistically significant. Cox proportional hazard model analysis revealed that patients in the high-MLR (HR 2.69, 95% CI: 1.67–4.33) and high-NLR (HR 2.28, 95% CI: 1.40–3.68) groups had significantly greater all-cause mortality than did those in the low- and medium-MLR groups. Even after accounting for factors such as age, sex, race/ethnicity, marital status, education, income, diabetes status, hypertension status, smoking status, alcohol consumption status, and BMI, the observed differences in all-cause mortality among participants with RA remained significant. Furthermore, the mortality rate increased as the NLR increased. Moreover, we analyzed the relationship between the Cox proportional hazards model, PLR, and RA mortality. However, a significant difference was not found in mortality rates among RA patients in different PLR groups, both in the unadjusted crude model and in the model with adjusted covariates.

**TABLE 1:** Association of complete blood cell (CBC)-derived indicators with mortality risk in participants with rheumatoid arthritis (RA).

	Crude model		Model 1		Model 2		Model 3	
Characteristic	HR (95% CI)	*P*	HR (95% CI)	*P*	HR (95% CI)	*P*	HR (95% CI)	*P*
**SIRI**								
Low	1 (ref)	–	1 (ref)	–	1 (ref)	–	1 (ref)	–
Medium	1.52 (0.90, 2.56)	0.11	1.46 (0.89, 2.41)	0.14	1.46 (0.88, 2.43)	0.14	1.36 (0.82, 2.28)	0.24
High	2.58 (1.61, 4.12)	**< 0.001**	2.17 (1.35, 3.47)	**0.001**	2.01 (1.22, 3.30)	**0.01**	1.87 (1.12, 3.13)	**0.02**
P for trend	–	**< 0.001**	–	**<0.001**	–	**0.01**	–	**0.01**
**SII**								
Low	1 (ref)	–	1 (ref)	–	1 (ref)	–	1 (ref)	–
Medium	1.03 (0.70, 1.53)	0.87	1.03 (0.70, 1.52)	0.89	1.09 (0.72, 1.66)	0.68	1.00 (0.64, 1.56)	0.99
High	1.45 (1.04, 2.02)	**0.03**	1.46 (1.07, 2.00)	**0.02**	1.35 (0.97, 1.86)	0.07	1.26 (0.92, 1.72)	0.15
P for trend	–	**0.02**	–	**0.01**	–	0.07	–	0.12
**NLR**								
Low	1 (ref)	–	1 (ref)	–	1 (ref)	–	1 (ref)	–
Medium	1.36 (0.83, 2.23)	0.23	1.27 (0.79, 2.04)	0.32	1.34 (0.82, 2.18)	0.24	1.26 (0.77, 2.06)	0.36
High	2.28 (1.40, 3.68)	**< 0.001**	1.95 (1.21, 3.13)	**0.01**	1.88 (1.15, 3.08)	**0.01**	1.79 (1.10, 2.92)	**0.02**
P for trend	–	**< 0.001**	–	**0.003**	–	**0.01**	–	**0.01**
**PLR**								
Low	1	–	1	–	1	–	1	–
Medium	0.92 (0.57, 1.48)	0.73	0.93 (0.57, 1.49)	0.75	1.05 (0.66, 1.67)	0.84	1.06 (0.66, 1.68)	0.82
High	1.34 (0.89, 2.02)	0.17	1.26 (0.84, 1.89)	0.27	1.24 (0.84, 1.83)	0.27	1.32 (0.90, 1.93)	0.15
P for trend	–	0.16	–	0.25	–	0.27	–	0.14
**MLR**								
Low	1 (ref)	–	1 (ref)	–	1 (ref)	–	1 (ref)	–
Medium	1.66 (1.00, 2.76)	0.05	1.41 (0.84, 2.36)	0.19	1.31 (0.79, 2.17)	0.29	1.43 (0.87, 2.37)	0.16
High	2.69 (1.67, 4.33)	**< 0.001**	1.98 (1.21, 3.22)	**0.01**	1.75 (1.07, 2.85)	**0.02**	1.88 (1.17, 3.02)	**0.01**
P for trend	–	**< 0.001**	–	**0.004**	–	**0.02**	–	**0.01**

Model 1: adjusted for age, gender. Model 2: additional adjustment for race/ethnicity, marital status, education, and PIR. Model 3: additional adjustment for diabetes status, hypertension status, smoking status, alcohol use status, and BMI. The bold values indicate statistical significance.

**FIGURE 2:**
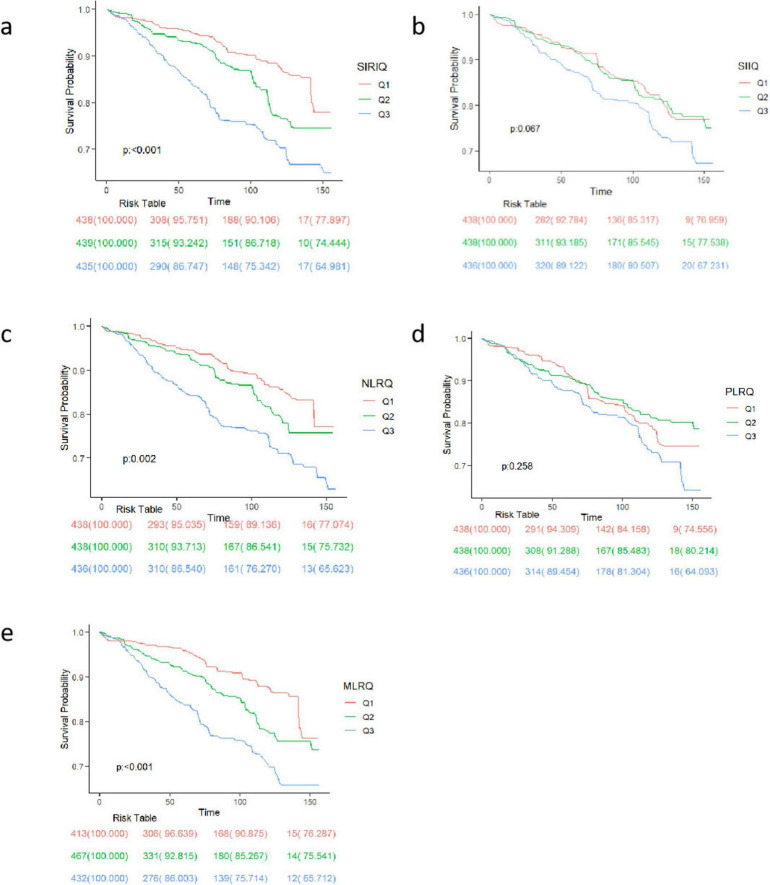
Mortality rates among participants with RA according to Kaplan–Meier curves. Kaplan–Meier curves stratified by **(a)** SII, **(b)** NLR, **(c)** NLR, **(d)** PLR, **(e)** MLR. The figure shows the survival probability trends of three groups (Q1, Q2, Q3) at different time points. The *p*-value indicates the statistical significance of survival differences among groups. The risk table below lists the remaining number of individuals and corresponding survival rates for each group at each time point.

As shown by Kaplan-Meier analysis results in Figure 2, CBC-derived indicators were significantly associated with higher mortality rates for individuals in the high SIRI, high NLR, and high MLR groups than in low SIRI, low NLR, and low MLR groups in RA patients (*p* < 0.001 for all). However, the survival rates among different SII and PLR groups did not differ significantly.

### 3.3 The non-linear correlation between mortality and CBC-derived indicators

**FIGURE 3:**
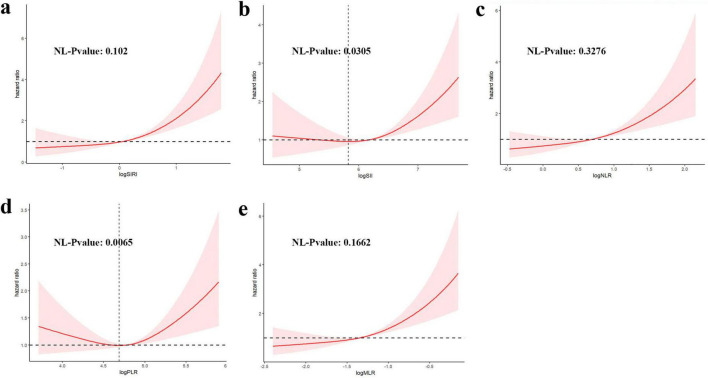
Restricted cubic spline (RCS) illustrating the correlation between indicators [**(a)** SIRI, **(b)** SII, **(c)** NLR, **(d)** PLR, **(e)** MLR] and mortality among participants with RA.

Figure 3 shows RCS curve of the association CBC-derived indicators with the risk of mortality rate in RA participants. A linear correlation between the SIRI, NLR, and MLR and all-cause mortality in RA patients is shown in Figures 3a, c, e. An elevated SIRI is connected to increased all-cause mortality in individuals with RA, however, there was no statistically significant non-linear correlation. In contrast, Figure 3b demonstrates a non-linear relationship between SII and all-cause mortality. Specifically, When the log of SII exceeds 5.82, all-cause mortality increases sharply with increasing SII (non-linear *P*-value = 0.03). Figure 3d reveals a non-linear correlation between the PLR and mortality in patients with RA. When the log PLR exceeds 4.68, the overall mortality rate of patients with RA decreased as the PLR increased. Conversely, while when the log PLR exceeds 4.68, the overall mortality increases with the PLR increased (non-linear *P*-value < 0.001).

### 3.4 Association between CBC characteristics and inflammatory indicators obtained from CBC

**FIGURE 4:**
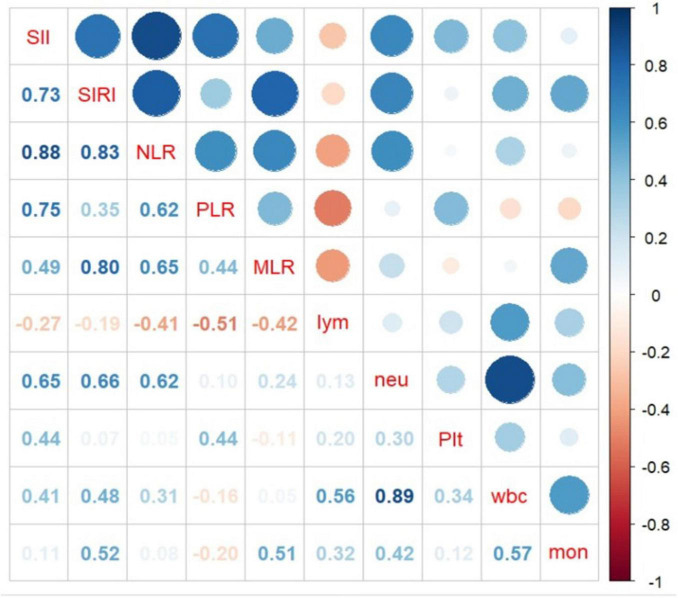
Association between complete blood cell (CBC) characteristics and inflammatory biomarkers obtained from complete blood cell (CBC).

The relationship between CBC parameters and indicators are depicted in Figure 4. The results indicate that neutrophil count and SIRI and SII are statistically significantly correlated with 0.66 and 0.65 correlation coefficients, respectively.

## 4 Discussion

Over a period of 78 months, a cohort of 1,314 adult RA patients was examined to assess the potential association between inflammatory indicators derived from CBC and mortality risk. The relationship between inflammatory indicators derived from CBC and all-cause mortality has been infrequently documented in the context of autoimmune diseases. However, recent years have witnessed a growing interest in the application of CBC-derived inflammatory indicators for evaluating disease activity in various autoimmune conditions, including systemic lupus erythematosus (23, 24), Sjogren’s syndrome (25), anti-synthetase syndrome (26), and Behcet’s disease (27), as well as in spondylarthritis (28) and systemic sclerosis (29). Furthermore, numerous studies also have identified associations between CBC-derived inflammatory indicators and disease complications, such as lupus nephritis (30), peripheral neuropathy in Sjogren’s syndrome (31), and ocular features of Behcet’s disease patients (32). CBC-derived inflammatory indicators are simple and easily available biomarkers of systemic inflammation, initially recognized for their highly sensitive as inflammatory indicators in the fields of oncology, particularly in gastric cancer (33), breast cancer (34), small cell lung cancer (35), and prostate cancer (36). CBC-derived inflammatory indicators have been validated as independent prognostic risk factors in participants with tumors, contributing to the prognostic assessment of disease treatment outcomes. These findings have inspired us to investigate the associations of inflammatory indicators with the prognosis and mortality of participants with RA. For the first time, our findings suggest that RA patients with high levels of SIRI, NLR, and MLR have a greater risk of mortality. K-M curves and RCS analyses demonstrated significant reductions and linear positive associations in survival probabilities for those with higher SIRI, NLR and MLR. These findings suggest that the SIRI, NLR, and MLR, which are widely available and inexpensive indicators are linked to increased risks of mortality in patients with RA.

Data from the Baltimore Longitudinal Study of Aging (BLSA) further corroborate these findings, demonstrating that higher white blood cell counts were associated with increased mortality (37). Additionally, NLR and MLR have been identified as leukocyte-derived ratios predictive of multimorbidity and mortality within the BLSA cohort (38). Recently, the InCHIANTI study found that the NLR and MLR were reliable indicators of healthy aging and predicted changes physical resilience and mortality (39–41). Similarly, the Rotterdam Study has established an association between the NLR and mortality within the general population (42). Obviously, leukocyte derived inflammatory indicators have become a global research hotspot, and their significances in monitoring chronic diseases need to be explored further.

Inflammatory indicators are derived from the combination of neutrophils, lymphocytes, monocytes, or platelets obtained through routine blood tests. The NLR is calculated as the ratio of neutrophils to lymphocytes, reflecting the activity of cells within both the innate and adaptive immune systems. Neutrophils can produce a variety of lyases, cytokines and oxygen free radicals, which characterize the innate system as the first line of defense. In contrast, the continuous accumulation of lymphocytes in inflamed joints leads to a reduction in peripheral blood lymphocyte counts in RA patients. Lymphocytes represent the adaptive immune response. Therefore, routine blood tests are gaining attention due to decreasing peripheral blood lymphocytes and increasing neutrophil counts. Monocytes exert a regulatory influence on the immune system, engage in cytokine synthesis, and can cause bone degradation in RA. The MLR is calculated by dividing monocyte count by lymphocyte count. The SIRI reflects the interactions among neutrophils, monocytes, and lymphocytes, illustrating the intricate interplay and potential synergistic effects among these cell types. The evaluation and prognosis of rheumatoid arthritis patients involve the SIRI, NLR, and MLR due to a series of alterations in neutrophils, lymphocytes, and monocytes. The limits of CBC-derived inflammatory indicators in distinguishing patients with RA from those with other rheumatic illnesses are evident (43). However, in persons with RA, the peripheral blood contains more neutrophils and monocytes, while lymphocytes are reduced. Our research suggests that these indicators play a significant role in assessing overall mortality in individuals with RA. CBC-derived inflammatory indicators represent novel, accessible, and non-invasive tools of notable importance. Their efficacy and simplicity facilitate rheumatologists in assessing patient prognosis.

Previous researches have revealed a correlation between CBC-derived inflammatory indicators and RA. These biomarkers serve prognostic functions in rheumatic illnesses and can be employed to evaluate disease progression (15, 44). While ultrasound and magnetic resonance imaging can detect joint inflammation, these methods are complex, occasionally difficult to access, and may require specialized techniques. Conversely, although blood cell-derived indicators do not support directly diagnosis of RA, they may reflect chronic inflammatory burden associated with rheumatic diseases (43). Furthermore, correlations have been established between complex blood inflammation markers and cardiovascular risk factors, as well as subclinical atherosclerosis, in individuals diagnosed with RA (45). Numerous studies have documented the ability of CBC-derived inflammatory indicators to identify disease activity and predict disease status of patients with RA. However, few studies have reported their relationship with all-cause mortality in this population.

A measure of all-cause mortality can be employed to understand disease prevalence and assess the effectiveness of health management strategies. Therefore, it is essential to identify readily available biomarkers linked to overall mortality across various conditions. Numerous studies have documented correlations between one or two inflammatory biomarkers generated from CBCs to assess their connection with death from any cause. For instance, the SII and SIRI have been strongly related to cardiovascular mortality and all-cause mortality events, and more attention should be given to systemic immune inflammation to provide new insights into prevention (46). Moreover, the SIRI and SII independently predict all-cause and cardiovascular disease (CVD) mortality events in obese individuals. Notably, SIRI exhibits a significantly greater predictive value than SII, suggesting that it is a more meaningful marker of inflammation (47). In patients diagnosed with ANCA-associated vasculitis (AAV), the SIRI at the time of diagnosis predicts all-cause mortality during follow-up (48). Similarly, in patients with hypertension, an elevated SIRI is also correlated with increased all-cause mortality and CVD mortality (49). Additionally, the NLR has been associated with all-cause mortality with stage five chronic kidney disease (50) and with cardiovascular mortality risk in maintenance hemodialysis patients (51) and patients with RA (16). Researchers have also found a significant association between a high MLR and an elevated risk of death from chronic kidney disease and type 2 diabetes mellitus within 90 days (52). However, our study provided new strategies and supporting materials that highlight the significance of the SIRI, NLR, and MLR as independent predictors of mortality related to RA.

Patients with RA are characterized by the production of autoantibodies and systemic inflammation, which lead to the activation and release of immune cell, including neutrophils, lymphocytes and monocytes (53). Therefore, three kinds of cell counts are invaluable for understanding the inflammatory state and immune response reflected in RA. Several studies have suggested that the immunological equilibrium resulting from the interplay of leukocytes, synovial fibroblasts, chondrocytes, and osteoclasts may have a significant effect on the development of RA. One hypothesis posits that neutrophils, because their pronounced cytotoxicity, may generate degradation enzymes and reactive oxygen species that could serve as antigens in the autoimmune process, thereby affecting the underlying mechanism of the autoimmune response (54–57). An alternative hypothesis proposes that the presence of inflammation in individuals with RA may stimulate the activation of apoptotic cytokines and granulocyte colony-stimulating factor, which could promote the activation and proliferation of neutrophils, initiating the immune response through a positive feedback loop. This mechanism can ultimately contribute to the onset and progression of RA (58). Lymphocytes are a vital component of the host immune system and serve as protective factor for prognosis in RA patients (59). Moreover, monocytes have a significant impact on the pathogenesis of RA. Osteoclasts, characterized as large multinucleated cells, originate from the monocyte–macrophage lineage. Upon specific stimulation, circulating monocytes migrate to designated sites within the bone, where they fuse with fully developed multinucleated osteoclasts and actively engage in the process of bone resorption. The process of transition from monocytes to osteoclasts is essential for joint deterioration, contributing to both in inflammation exacerbation and bone degradation in RA, despite the presence of osteoclast precursors other than monocytes (60–62). High values of the SIRI, NLR and MLR reflect both the ability of monocytes and neutrophils to mediate a strong proinflammatory response and the ability of lymphocytes to mediate a weak or inhibited anti-inflammatory response. Our study indicates that increased SIRI, NLR, and MLR are associated with greater risks of RA-related mortality. Attenuating immune inflammation emerges as a promising strategy for retarding RA progression and limiting adverse outcomes.

Our article has several advantages. Firstly, it features a substantial sample size with a representative sample selection. This study is the first to identify associations between inflammatory indicators derived from CBCs in patients suffering from RA and all-cause mortality, a large sample size was used. The analysis employed a weighted logistic regression model, adjusting for other covariates. Secondly, prior to this study, no research had comprehensively examined the relationship between inflammatory indicators derived from CBC and all-cause mortality in RA. Until now, there had been no article on the correlation between MLR and all-cause mortality in RA. It has been suggested that the MLR can serve as a complementary diagnostic indicator for the diagnosis of RA (18) and associated with disease activity and specific clinical features of RA (63). Consequently, our conclusions are more accurate and reliable. Lastly, the non-linear relationship is explored by using restricted cubic splines and smooth curve fitting, after which the inflection points are further calculated. A notable observation in our study is that the risk of mortality in RA patients increased over time, consistent with our understanding of disease-related harm. Therefore, our findings demonstrate enhanced greater persuasiveness, comprehensiveness, and robust documentation.

Meanwhile, some inevitable limitations should be acknowledged in our study. First and foremost, the cross-sectional study design was unable to determine a clear causal association between inflammatory indicators derived from CBCs in patients with RA and all-cause mortality. Moreover, CBC-derived inflammatory biomarkers were measured at a single timepoint and most likely did not reflect changes in time or intervention. Finally, given that the population in the present study was drawn from a representative sample within the United States population, a replication of our results in other racial groups is necessary.

## 5 Conclusion

This study suggested that increased SIRI, NLR, and MLR were associated with greater risks of RA-related mortality and can be used to measure pathological innate inflammation and protective adaptive immunity. In addition to helping to discover their potential utility in predicting RA outcomes, these findings also provide rheumatologists with guidance on disease management.

## Data Availability

The original contributions presented in this study are included in this article/Supplementary material, further inquiries can be directed to the corresponding authors.
