# Supplementary material for: Correlation between CBC-derived inflammatory indicators and all-cause mortality with rheumatoid arthritis: a population-based study
Source: Front Med (Lausanne). 2025 Jun 10;12:1538710. doi: 10.3389/fmed.2025.1538710 (PMC12185416; doi:10.3389/fmed.2025.1538710)
Supplement: Supplementary file 1 [file Table_1.docx]

Table S1. Basic characteristics of participants with RA based on CBC-derived inflammatory indicators.

a.

| SIRI | Low:0.14-0.81 | Medium:0.81-1.38 | High:1.38-24.6 | *p-value* |
| --- | --- | --- | --- | --- |
| SII | 331.27± 8.30 | 505.19±14.19 | 869.67±34.77 | < 0.001 |
| NLR | 1.41±0.03 | 2.08±0.04 | 3.47±0.10 | < 0.001 |
| PLR | 111.11±2.78 | 125.73±2.88 | 149.48±4.89 | < 0.001 |
| MLR | 0.20±0.00 | 0.27±0.01 | 0.41±0.01 | < 0.001 |
| Lymphocyte, 10^3^/uL | 2.34±0.06 | 2.10±0.04 | 1.92±0.05 | < 0.001 |
| Neutrophils, 10^3^/uL | 3.04±0.04 | 4.12±0.08 | 5.96±0.13 | < 0.001 |
| Platelet, 10^3^/uL | 238.25±5.14 | 243.24±4.27 | 253.18±5.42 | 0.12 |
| White blood cell, 10^3^/uL | 6.03±0.09 | 7.03±0.10 | 8.88±0.17 | < 0.001 |
| Monocyte, 10^3^/uL | 0.44±0.01 | 0.54±0.01 | 0.71±0.02 | < 0.001 |
| Gender, % |  |  |  | 0.004 |
| Female | 67.06 | 61.28 | 49.62 |  |
| Male | 32.94 | 38.72 | 50.38 |  |
| Age, % |  |  |  | 0.001 |
| <60 | 61.05 | 59.81 | 44.76 |  |
| >=60 | 38.95 | 40.19 | 55.24 |  |
| BMI, % |  |  |  | 0.79 |
| Normal | 20.98 | 23.93 | 21.86 |  |
| Overweight/Obesity | 79.02 | 76.07 | 78.14 |  |
| Race/ethnicity |  |  |  | < 0.001 |
| Mexican American | 6.86 | 7.60 | 6.60 |  |
| Non-Hispanic Black | 25.45 | 14.02 | 9.66 |  |
| Non-Hispanic White | 52.19 | 69.79 | 75.79 |  |
| Other | 15.50 | 8.59 | 7.95 |  |
| PIR, % |  |  |  | 0.83 |
| Higher income | 33.24 | 33.62 | 29.85 |  |
| Lower income | 33.32 | 30.72 | 32.42 |  |
| Moderate income | 33.44 | 35.66 | 37.73 |  |
| Education level, % |  |  |  | 0.35 |
| College or above | 51.63 | 56.50 | 48.07 |  |
| High school or equivalent | 24.42 | 23.49 | 29.47 |  |
| Less than high school | 23.95 | 20.01 | 22.46 |  |
| Alcohol using status, % |  |  |  | 0.59 |
| Ever/current | 86.25 | 88.81 | 86.66 |  |
| Never | 13.75 | 11.19 | 13.34 |  |
| Smoking status, % |  |  |  | 0.46 |
| Former | 31.37 | 28.75 | 32.18 |  |
| Never | 44.28 | 48.64 | 39.75 |  |
| Now | 24.36 | 22.61 | 28.08 |  |
| Diabetes, % |  |  |  | 0.46 |
| Yes | 23.29 | 28.04 | 26.49 |  |
| No | 76.71 | 71.96 | 73.51 |  |
| Hypertension, % |  |  |  | < 0.001 |
| No | 44.47 | 43.99 | 28.75 |  |
| Yes | 55.53 | 56.01 | 71.25 |  |
| Marital status, % |  |  |  | 0.37 |
| Married/cohabiting | 58.27 | 64.90 | 59.85 |  |
| Widowed/divorced/ separated | 41.73 | 35.10 | 40.15 |  |

b.

| SII | Low:42.42-375.96 | | Medium:375.96-607.47 | | High:607.47-6297.6 | | *p-value* |
| --- | --- | --- | --- | --- | --- | --- | --- |
| SIRI | | 0.75±0.02 | | 1.16±0.03 | | 2.22±0.09 | < 0.001 |
| NLR | | 1.39±0.03 | | 2.05±0.03 | | 3.64±0.09 | < 0.001 |
| PLR | | 93.30±1.82 | | 120.07±2.09 | | 174.65±5.46 | < 0.001 |
| MLR | | 0.24±0.01 | | 0.28±0.01 | | 0.37±0.01 | < 0.001 |
| Lymphocyte,10^3^/uL | | 2.38±0.05 | | 2.16±0.06 | | 1.80±0.05 | < 0.001 |
| Neutrophils, 10^3^/uL | | 3.14±0.06 | | 4.22±0.08 | | 5.94±0.14 | < 0.001 |
| Platelet, 10^3^/uL | | 205.86±3.45 | | 243.56±3.63 | | 284.65±6.31 | < 0.001 |
| White blood cell,10^3^/L | | 6.28±0.10 | | 7.20±0.14 | | 8.64±0.19 | < 0.001 |
| Monocyte, 10^3^/uL | | 0.54±0.01 | | 0.56±0.01 | | 0.61±0.02 | 0.003 |
| Gender, % | |  | |  | |  | 0.33 |
| Female | | 55.04 | | 59.23 | | 62.16 |  |
| Male | | 44.96 | | 40.77 | | 37.84 |  |
| Age, % | |  | |  | |  | 0.39 |
| <60 | | 56.13 | | 57.16 | | 51.13 |  |
| >=60 | | 43.87 | | 42.84 | | 48.87 |  |
| BMI, % | |  | |  | |  | 0.95 |
| Normal | | 21.70 | | 22.14 | | 23.05 |  |
| Overweight/Obesity | | 78.30 | | 77.86 | | 76.95 |  |
| Race/ethnicity | |  | |  | |  | < 0.001 |
| Mexican American | | 7.57 | | 6.30 | | 7.24 |  |
| Non-Hispanic Black | | 22.74 | | 13.14 | | 12.35 |  |
| Non-Hispanic White | | 56.48 | | 71.69 | | 70.92 |  |
| Other | | 13.21 | | 8.87 | | 9.50 |  |
| PIR, % | |  | |  | |  | 0.31 |
| Higher income | | 34.30 | | 35.45 | | 26.69 |  |
| Lower income | | 32.27 | | 30.12 | | 34.05 |  |
| Moderate income | | 33.43 | | 34.43 | | 39.26 |  |
| Education level, % | |  | |  | |  | 0.05 |
| College or above | | 52.33 | | 58.12 | | 45.38 |  |
| High school or equivalent | | 24.25 | | 22.56 | | 30.95 |  |
| Less than high school | | 23.42 | | 19.32 | | 23.67 |  |
| Alcohol using status, % | |  | |  | |  | 0.7 |
| Ever/current | | 86.41 | | 88.44 | | 86.86 |  |
| Never | | 13.59 | | 11.56 | | 13.14 |  |
| Smoking status, % | |  | |  | |  | 0.36 |
| Former | | 32.52 | | 29.17 | | 30.75 |  |
| Never | | 45.47 | | 47.27 | | 39.63 |  |
| Now | | 22.01 | | 23.56 | | 29.62 |  |
| Diabetes, % | |  | |  | |  | 0.61 |
| Yes | | 24.60 | | 25.38 | | 28.13 |  |
| No | | 75.40 | | 74.62 | | 71.87 |  |
| Hypertension, % | |  | |  | |  | 0.01 |
| No | | 42.99 | | 43.39 | | 29.74 |  |
| Yes | | 57.01 | | 56.61 | | 70.26 |  |
| Marital status, % | |  | |  | |  | 0.87 |
| Married/cohabiting | | 60.55 | | 62.61 | | 60.02 |  |
| Widowed/divorced/separated | | 39.45 | | 37.39 | | 39.98 |  |

c.

| NLR | Low:0.19-1.64 | | Medium:1.64-2.47 | High:2.47-24.60 | *p-value* |
| --- | --- | --- | --- | --- | --- |
| SIRI | | 0.67±0.02 | 1.18±0.03 | 2.31±0.08 | < 0.001 |
| SII | | 303.31± 7.39 | 494.82± 9.28 | 965.04±35.38 | < 0.001 |
| PLR | | 100.61±2.59 | 120.91±2.28 | 168.23±5.35 | < 0.001 |
| MLR | | 0.22±0.01 | 0.28±0.00 | 0.39±0.01 | < 0.001 |
| Lymphocyte, 10^3^/uL | | 2.55±0.05 | 2.14±0.04 | 1.64±0.04 | < 0.001 |
| Neutrophils, 10^3^/uL | | 3.14±0.05 | 4.32±0.09 | 5.89±0.13 | < 0.001 |
| Platelet, 10^3^/uL | | 239.89±4.83 | 244.45±4.23 | 251.48±5.86 | 0.28 |
| White blood cell, 10^3^/uL | | 6.47±0.10 | 7.30±0.15 | 8.39±0.18 | < 0.001 |
| Monocyte, 10^3^/uL | | 0.54±0.01 | 0.58±0.01 | 0.60±0.02 | 0.001 |
| Gender, % | |  |  |  | 0.47 |
| Female | | 62.31 | 58.20 | 56.23 |  |
| Male | | 37.69 | 41.80 | 43.77 |  |
| Age, % | |  |  |  | 0.002 |
| <60 | | 61.72 | 56.49 | 46.18 |  |
| >=60 | | 38.28 | 43.51 | 53.82 |  |
| BMI, % | |  |  |  | 0.88 |
| Normal | | 20.98 | 22.95 | 22.90 |  |
| Overweight/Obesity | | 79.02 | 77.05 | 77.10 |  |
| Race/ethnicity | |  |  |  | < 0.001 |
| Mexican American | | 6.10 | 7.37 | 7.54 |  |
| Non-Hispanic Black | | 23.42 | 13.14 | 11.60 |  |
| Non-Hispanic White | | 56.05 | 71.62 | 71.48 |  |
| Other | | 14.43 | 7.87 | 9.38 |  |
| PIR, % | |  |  |  | 0.36 |
| Higher income | | 33.81 | 35.60 | 26.77 |  |
| Lower income | | 32.37 | 30.55 | 33.58 |  |
| Moderate income | | 33.82 | 33.86 | 39.65 |  |
| Education level, % | |  |  |  | 0.11 |
| College or above | | 54.40 | 56.04 | 45.28 |  |
| High school or equivalent | | 23.11 | 23.28 | 31.53 |  |
| Less than high school | | 22.49 | 20.68 | 23.19 |  |
| Alcohol using status, % | |  |  |  | 0.3 |
| Ever/current | | 87.77 | 88.85 | 85.05 |  |
| Never | | 12.23 | 11.15 | 14.95 |  |
| Smoking status, % | |  |  |  | 0.82 |
| Former | | 28.22 | 31.26 | 32.74 |  |
| Never | | 46.07 | 44.97 | 41.36 |  |
| Now | | 25.71 | 23.77 | 25.91 |  |
| Diabetes, % | |  |  |  | 0.35 |
| Yes | | 22.92 | 26.84 | 28.27 |  |
| No | | 77.08 | 73.16 | 71.73 |  |
| Hypertension, % | |  |  |  | 0.01 |
| No | | 41.82 | 43.90 | 29.94 |  |
| Yes | | 58.18 | 56.10 | 70.06 |  |
| Marital status, % | |  |  |  | 0.45 |
| Married/cohabiting | | 59.21 | 64.43 | 59.28 |  |
| Widowed/divorced  /separated | | 40.79 | 35.57 | 40.72 |  |

d.

| PLR | Low:9.41-101.34 | | Medium:101.34-140.44 | | High:140.44-778.33 | *p-value* |
| --- | --- | --- | --- | --- | --- | --- |
| SIRI | | 1.10±0.05 | | 1.24±0.08 | 1.83±0.09 | < 0.001 |
| SII | | 360.71±10.85 | | 507.63±16.22 | 909.15±35.46 | < 0.001 |
| NLR | | 1.73±0.05 | | 2.11±0.06 | 3.33±0.10 | < 0.001 |
| MLR | | 0.24±0.01 | | 0.28±0.01 | 0.37±0.01 | < 0.001 |
| Lymphocyte, 10^3^/uL | | 2.72±0.05 | | 2.05±0.04 | 1.53±0.03 | < 0.001 |
| Neutrophils, 10^3^/uL | | 4.48±0.12 | | 4.20±0.13 | 4.68±0.13 | 0.03 |
| Platelet, 10^3^/uL | | 212.74±3.85 | | 245.90±4.21 | 278.93±5.79 | < 0.001 |
| White blood cell, 10^3^/uL | | 8.07±0.15 | | 7.08±0.17 | 7.00±0.15 | < 0.001 |
| Monocyte, 10^3^/uL | | 0.62±0.02 | | 0.57±0.02 | 0.53±0.01 | < 0.001 |
| Gender, % | |  | |  |  | 0.03 |
| Female | | 51.79 | | 61.73 | 63.26 |  |
| Male | | 48.21 | | 38.27 | 36.74 |  |
| Age, % | |  | |  |  | 0.35 |
| <60 | | 58.65 | | 53.79 | 51.90 |  |
| >=60 | | 41.35 | | 46.21 | 48.10 |  |
| BMI, % | |  | |  |  | 0.36 |
| Normal | | 20.57 | | 20.80 | 25.76 |  |
| Overweight/Obesity | | 79.43 | | 79.20 | 74.24 |  |
| Race/ethnicity | |  | |  |  | 0.73 |
| Mexican American | | 7.92 | | 5.97 | 7.20 |  |
| Non-Hispanic Black | | 15.80 | | 15.69 | 16.33 |  |
| Non-Hispanic White | | 64.75 | | 69.45 | 65.44 |  |
| Other | | 11.53 | | 8.89 | 11.03 |  |
| PIR, % | |  | |  |  | 0.44 |
| Higher income | | 32.27 | | 35.79 | 28.10 |  |
| Lower income | | 32.63 | | 31.84 | 31.87 |  |
| Moderate income | | 35.09 | | 32.37 | 40.03 |  |
| Education level, % | |  | |  |  | 0.9 |
| College or above | | 52.73 | | 51.08 | 52.30 |  |
| High school or equivalent | | 26.65 | | 24.91 | 26.19 |  |
| Less than high school | | 20.62 | | 24.01 | 21.51 |  |
| Alcohol using status, % | |  | |  |  | 0.04 |
| Ever/current | | 90.41 | | 87.22 | 84.00 |  |
| Never | | 9.59 | | 12.78 | 16.00 |  |
| Smoking status, % | |  | |  |  | 0.19 |
| Former | | 31.89 | | 30.18 | 30.21 |  |
| Never | | 38.45 | | 45.19 | 49.03 |  |
| Now | | 29.65 | | 24.63 | 20.76 |  |
| Diabetes, % | |  | |  |  | 0.71 |
| Yes | | 27.74 | | 24.68 | 25.76 |  |
| No | | 72.26 | | 75.32 | 74.24 |  |
| Hypertension, % | |  | |  |  | 0.41 |
| No | | 42.25 | | 36.25 | 37.63 |  |
| Yes | | 57.75 | | 63.75 | 62.37 |  |
| Marital status, % | |  | |  |  | 0.65 |
| Married/cohabiting | | 60.11 | | 63.55 | 59.44 |  |
| Widowed/divorced  /separated | | 39.89 | | 36.45 | 40.56 |  |

e.

| MLR | Low:0.04-0.22 | | Medium:0.22-0.32 | | High:0.32-2.00 | *p-value* |
| --- | --- | --- | --- | --- | --- | --- |
| SIRI | | 0.72±0.02 | | 1.13±0.03 | 2.25±0.09 | < 0.001 |
| SII | | 429.63±16.14 | | 528.71±19.75 | 787.99±34.55 | < 0.001 |
| NLR | | 1.70±0.06 | | 2.11±0.05 | 3.26±0.10 | < 0.001 |
| PLR | | 103.85±2.98 | | 125.13±2.76 | 158.15±4.78 | < 0.001 |
| Lymphocyte, 10^3^/uL | | 2.62±0.07 | | 2.10±0.03 | 1.66±0.05 | < 0.001 |
| Neutrophils, 10^3^/uL | | 4.14±0.14 | | 4.24±0.09 | 4.94±0.14 | < 0.001 |
| Platelet, 10^3^/uL | | 253.09±5.80 | | 245.53±3.99 | 237.88±5.19 | 0.15 |
| White blood cell, 10^3^/uL | | 7.44±0.19 | | 7.17±0.12 | 7.57±0.20 | 0.16 |
| Monocyte, 10^3^/uL | | 0.45±0.01 | | 0.56±0.01 | 0.70±0.02 | < 0.001 |
| Gender, % | |  | |  |  | < 0.001 |
| Female | | 72.03 | | 55.20 | 50.72 |  |
| Male | | 27.97 | | 44.80 | 49.28 |  |
| Age, % | |  | |  |  | < 0.001 |
| <60 | | 64.35 | | 58.48 | 42.34 |  |
| >=60 | | 35.65 | | 41.52 | 57.66 |  |
| BMI, % | |  | |  |  | 0.19 |
| Normal | | 18.69 | | 21.78 | 26.13 |  |
| Overweight/Obesity | | 81.31 | | 78.22 | 73.87 |  |
| Race/ethnicity | |  | |  |  | < 0.001 |
| Mexican American | | 8.38 | | 6.48 | 6.34 |  |
| Non-Hispanic Black | | 18.97 | | 16.76 | 12.30 |  |
| Non-Hispanic White | | 56.42 | | 69.06 | 73.28 |  |
| Other | | 16.23 | | 7.69 | 8.08 |  |
| PIR, % | |  | |  |  | 0.69 |
| Higher income | | 34.48 | | 31.40 | 30.84 |  |
| Lower income | | 33.18 | | 32.84 | 30.39 |  |
| Moderate income | | 32.34 | | 35.77 | 38.76 |  |
| Education level, % | |  | |  |  | 0.88 |
| College or above | | 54.65 | | 50.46 | 51.27 |  |
| High school or equivalent | | 23.88 | | 27.25 | 26.34 |  |
| Less than high school | | 21.47 | | 22.30 | 22.40 |  |
| Alcohol using status, % | |  | |  |  | 0.11 |
| Ever/current | | 89.72 | | 87.72 | 84.57 |  |
| Never | | 10.28 | | 12.28 | 15.43 |  |
| Smoking status, % | |  | |  |  | 0.23 |
| Former | | 29.89 | | 31.54 | 30.75 |  |
| Never | | 39.12 | | 47.09 | 45.68 |  |
| Now | | 30.99 | | 21.37 | 23.57 |  |
| Diabetes, % | |  | |  |  | 0.56 |
| Yes | | 28.36 | | 24.49 | 25.56 |  |
| No | | 71.64 | | 75.51 | 74.44 |  |
| Hypertension, % | |  | |  |  | 0.01 |
| No | | 44.84 | | 41.30 | 30.43 |  |
| Yes | | 55.16 | | 58.70 | 69.57 |  |
| Marital status, % | |  | |  |  | 0.39 |
| Married/cohabiting | | 59.52 | | 64.41 | 59.06 |  |
| Widowed/divorced  /separated | | 40.48 | | 35.59 | 40.94 |  |

SIRI: Systemic inflammatory response index,

SII: Systemic immune-inflammation index,

NLR: Neutrophil-to-lymphocyte Ratio,

PLR: Platelet-to-lymphocyte ratio,

MLR: Monocyte-to-lymphocyte ratio,

BMI: Body Mass Index.
